# Case report: anti-GAD65 antibody-associated epilepsy as the primary manifestation of chronic graft-versus-host disease

**DOI:** 10.1007/s00277-026-06961-x

**Published:** 2026-03-30

**Authors:** Suge Yang, Pengchen Gu, Jingluan Tian, Xiaowen Tang, Qun Xue

**Affiliations:** 1 Department of Neurology, The First Affiliated Hospital of Soochow University, Suzhou, Jiangsu China; 2 Department of Hematology, The First Affiliated Hospital of Soochow University, Suzhou, Jiangsu China

**Keywords:** chronic graft-versus-host disease, anti-GAD65 antibody-associated epilepsy, immunoadsorption, Rituximab, Daratumumab, case report

## Abstract

Central nervous system involvement in chronic graft-versus-host disease (CNS-cGVHD) is rare. This case report describes a young man who developed anti-glutamic acid decarboxylase 65 (GAD65) antibody-associated epilepsy after undergoing matched sibling donor hematopoietic stem cell transplantation. The therapeutic approach included intensive immunotherapy with methylprednisolone, immunoadsorption, Rituximab, and low-dose Daratumumab, in combination with four antiepileptic drugs and mycophenolate mofetil. This regimen led to a marked reduction in anti-GAD65 titers and an improvement in the NHS3 seizure severity score, decreasing from 8 to 3. Additionally, a review of the literature on post-transplant autoimmune encephalitis is also provided.

## Introduction

Chronic graft-versus-host disease (cGVHD) represents a major complication of allogeneic hematopoietic stem cell transplantation (allo-HSCT), affecting 30–70% of recipients [1]. This condition arises when donor-derived immune cells attack host tissues, such as the skin, liver, and gastrointestinal tract [2, 3]. Central nervous system (CNS) involvement is uncommon and typically presents as cerebrovascular disease, demyelinating encephalopathy, or autoimmune encephalitis [4]. Pathological features include focal microglial activation and increased human leukocyte antigen-DR expression [5]. Seizures can occur as a clinical manifestation of CNS-cGVHD. While post-transplant epilepsy is commonly attributed to neurotoxicity, metabolic disturbances, cerebrovascular events, infection, or disease relapse, recent evidence suggests that some seizures of unknown etiology may be linked to cGVHD-mediated immune encephalitis [6, 7]. Reports of epilepsy linked to cGVHD-mediated autoimmune encephalitis remain scarce, with most cases demonstrating positive anti-N-methyl-D-aspartate receptor (NMDAR) and anti-GAD65 antibodies. Standard treatments include corticosteroids, intravenous immunoglobulin (IVIG), and antiepileptic drugs. Prognosis varies considerably, with a substantial risk of disability and mortality. Enhanced detection and management of these conditions are therefore critical. This report describes a rare case of anti-GAD65 antibody-associated epilepsy manifesting as CNS-cGVHD following allo-HSCT. The management strategy outlined may inform the treatment of similar cases.

## Case report

**Fig. 1:**
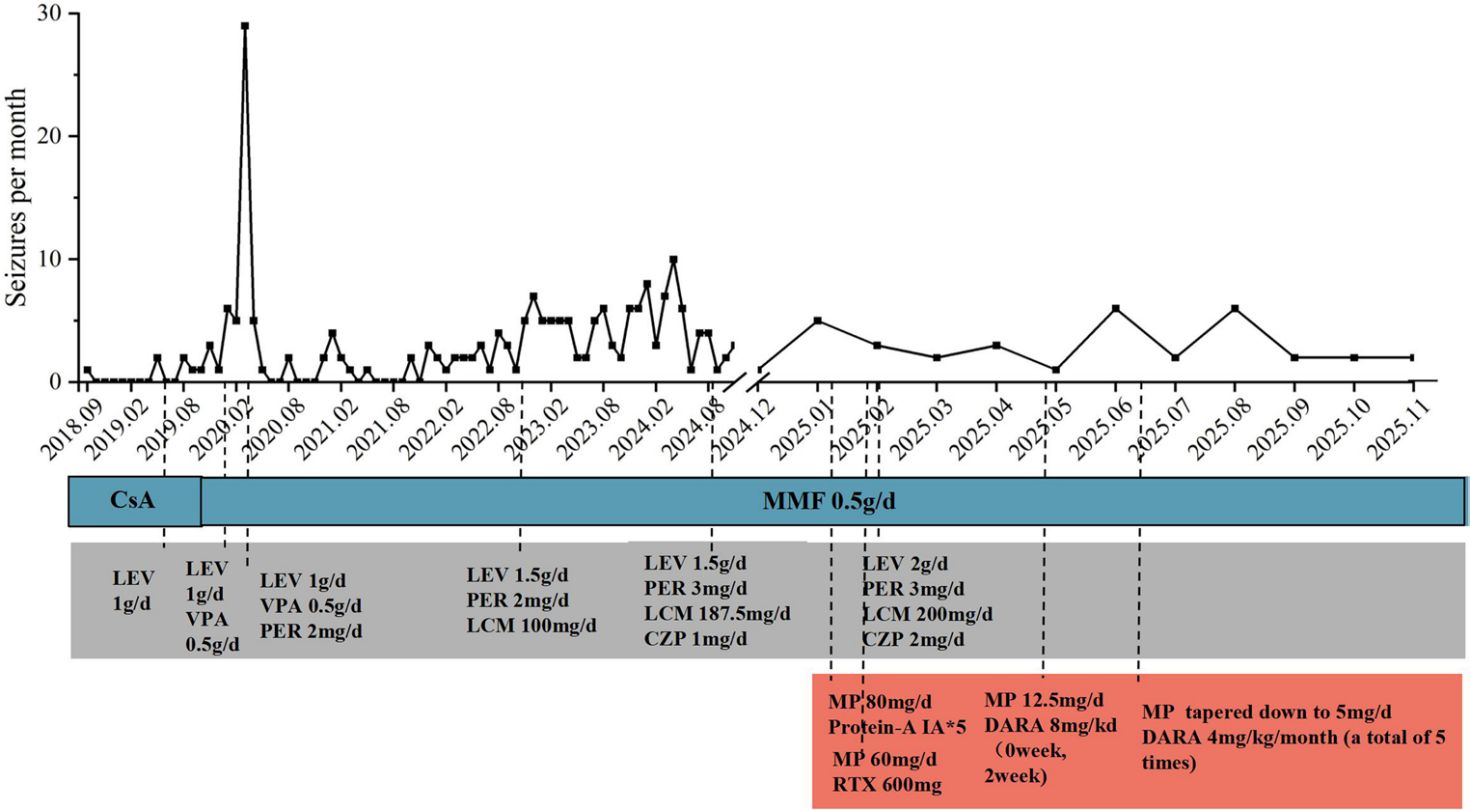
Timeline of seizure and clinical management. CsA: Cyclosporine; MMF: Mycophenolate Mofetil; LEV: Levetiracetam; VPA: Valproic acid sodium; PER: Perampanel; CZP: Clonazepam; LCM: Lacosamide; MP: Methylprednisolone; IA: Immunoadsorption; RTX: Rituximab; DARA: Daratumumab

A 29-year-old man presented in December 2024 with a seven-year history of recurrent seizures. The initial unprovoked seizure occurred in September 2018 and resolved spontaneously. By May 2019, seizure frequency increased to 0–2 episodes per month, manifesting as generalized tonic-clonic or focal seizures with impaired awareness. Previous evaluations at another institution, including cranial computed tomography (CT), contrast-enhanced magnetic resonance imaging (MRI), electroencephalography (EEG), and cerebrospinal fluid (CSF) analysis, were unremarkable. Levetiracetam was initiated and titrated to 750 mg twice daily. Despite treatment, seizure frequency progressively increased. EEG demonstrated prominent bilateral hemispheric paroxysmal delta waves and sharp waves. Multiple anti-seizure medications were subsequently trialed (see Fig. 1 for details). Currently, the patient experiences 1 to 7 seizures per month, including tonic, generalized tonic-clonic, and focal seizures with impaired awareness. Interictal symptoms include subjective cognitive decline, headache, anxiety, and depression. Neurological examination revealed no positive findings. Relevant scale scores were as follows: Mini-Mental State Examination (MMSE) 30/30, Montreal Cognitive Assessment (MoCA) 28/30 (university degree), Hamilton Anxiety Rating Scale (HAMA) 27, Hamilton Depression Rating Scale (HAMD) 30, and National Hospital Seizure Severity Scale (NHS3) 8.

The patient was diagnosed with acute lymphoblastic leukemia eleven years ago (in 2014). After conditioning with a modified BUCY regimen consisting of semustine, hydroxyurea, cytarabine, busulfan, and cyclophosphamide, he underwent allo-HSCT from a matched sibling donor. Bone marrow evaluation on day 14 post-transplant demonstrated remission. Long-term cyclosporine was administered to maintain immunosuppression (plasma concentration at 100–200 ng/mL), with cGVHD presenting solely as mild vitiligo. In September 2019, cyclosporine was replaced with mycophenolate mofetil (0.25 g twice daily). In December 2024, the patient developed abnormal liver function, with alanine aminotransferase (ALT) 216 U/L, aspartate aminotransferase (AST) 102 U/L, and gamma-glutamyl transferase (GGT) 114 U/L. Hepatitis B screening was negative, and abdominal CT was unremarkable. There was no family history of genetic diseases, nor any personal history of autoimmune diabetes, thyroid disorders, or other relevant conditions.

**Fig. 2:**
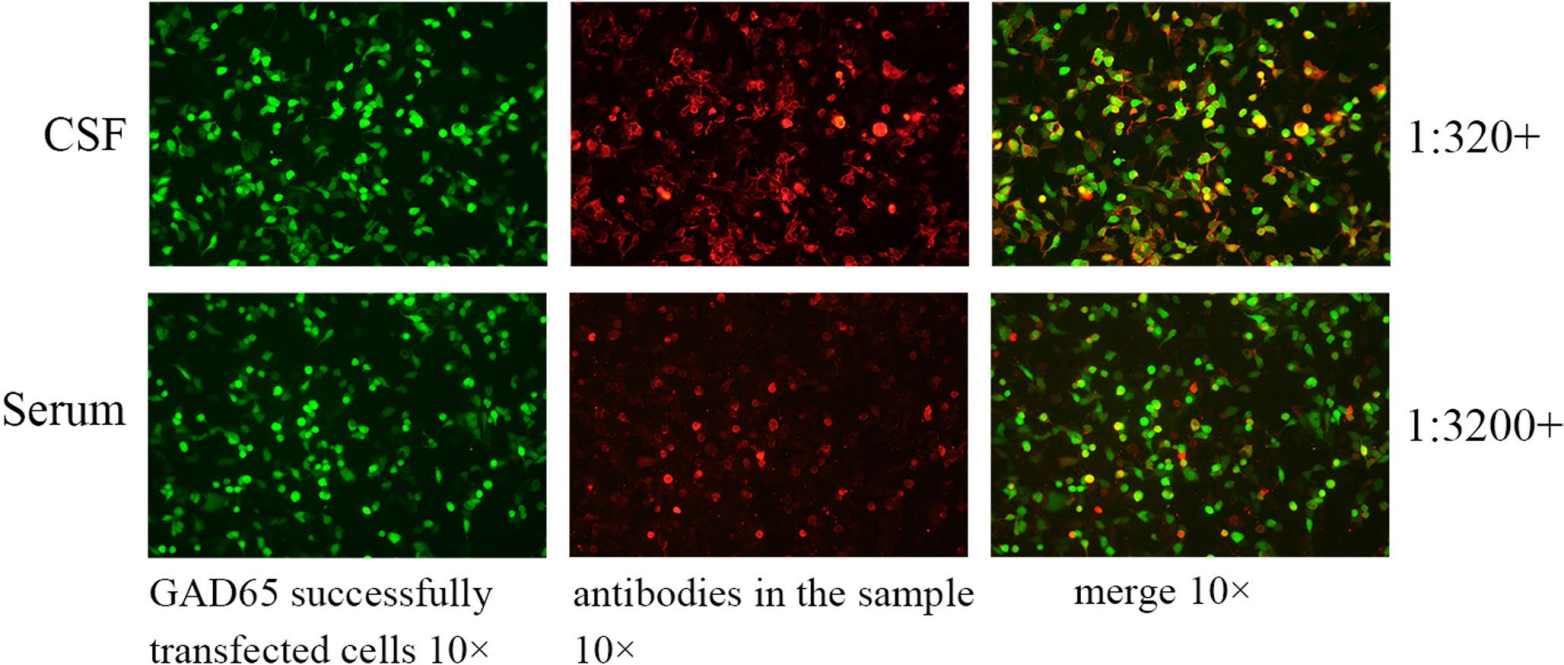
Detection of anti-GAD65 antibodies in the patient’s CSF and serum samples by CBA. (December 30, 2024; Xiansheng Medical Laboratory, China)

**Fig. 3:**
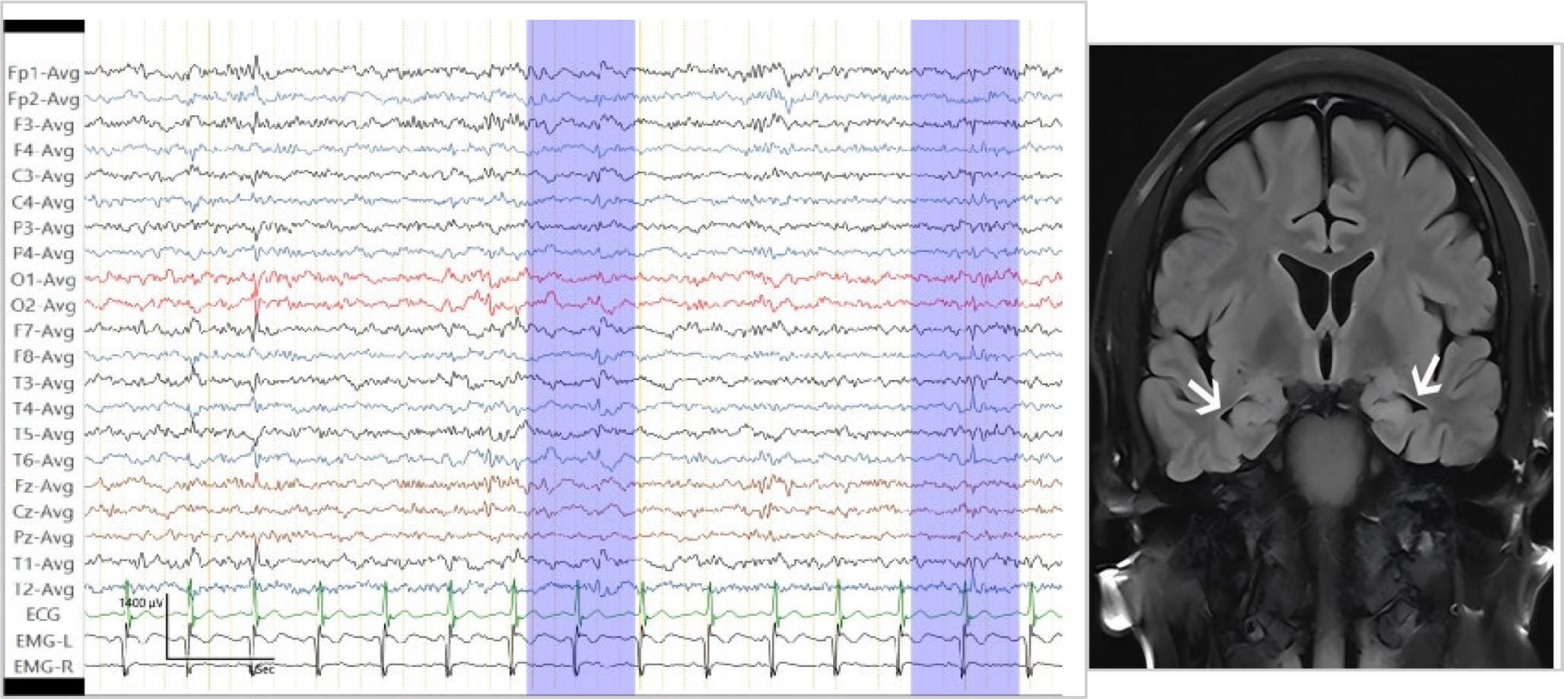
The patient’s EEG and coronal image of the hippocampus on brain MRI. Interictal EEG demonstrates frequent bilateral, scattered and paroxysmal, slow waves and sharp waves; the brain MRI image reveals mild hippocampal atrophy, as indicated by the white arrow

After the initial evaluation, a comprehensive screening for autoimmune antibodies was conducted, targeting those associated with autoimmune encephalitis, vasculitis, rheumatic diseases, and antiphospholipid syndrome, as well as tumor markers. CSF analysis showed normal routine and biochemical parameters, except for a positive oligoclonal band. Cell-based assays (CBA) detected anti-GAD65 antibody titers of 1:320 in the CSF and 1:3200 in the serum(Fig. 2), with NMDAR, LGI1, CASPR2, GABAbR, AMPAR1, AMPAR2, and MOG antibodies remaining negative. Tissue-based assays (TBA) confirmed the presence of antibodies in both serum and CSF. The serum anti-GAD antibody level, measured by chemiluminescence, was 712.68 IU/mL. Autoantibody screening was positive for antinuclear antibody (++) and anti-Ro-52 antibody (+++), while tumor marker screening was unremarkable. Brain MRI demonstrated no abnormal lesions other than mild hippocampal atrophy. Interictal EEG showed frequent, scattered, bilateral theta and sharp waves (Fig. 3). Collectively, these findings support a diagnosis of anti-GAD65 antibody-associated epilepsy.

**Fig. 4:**
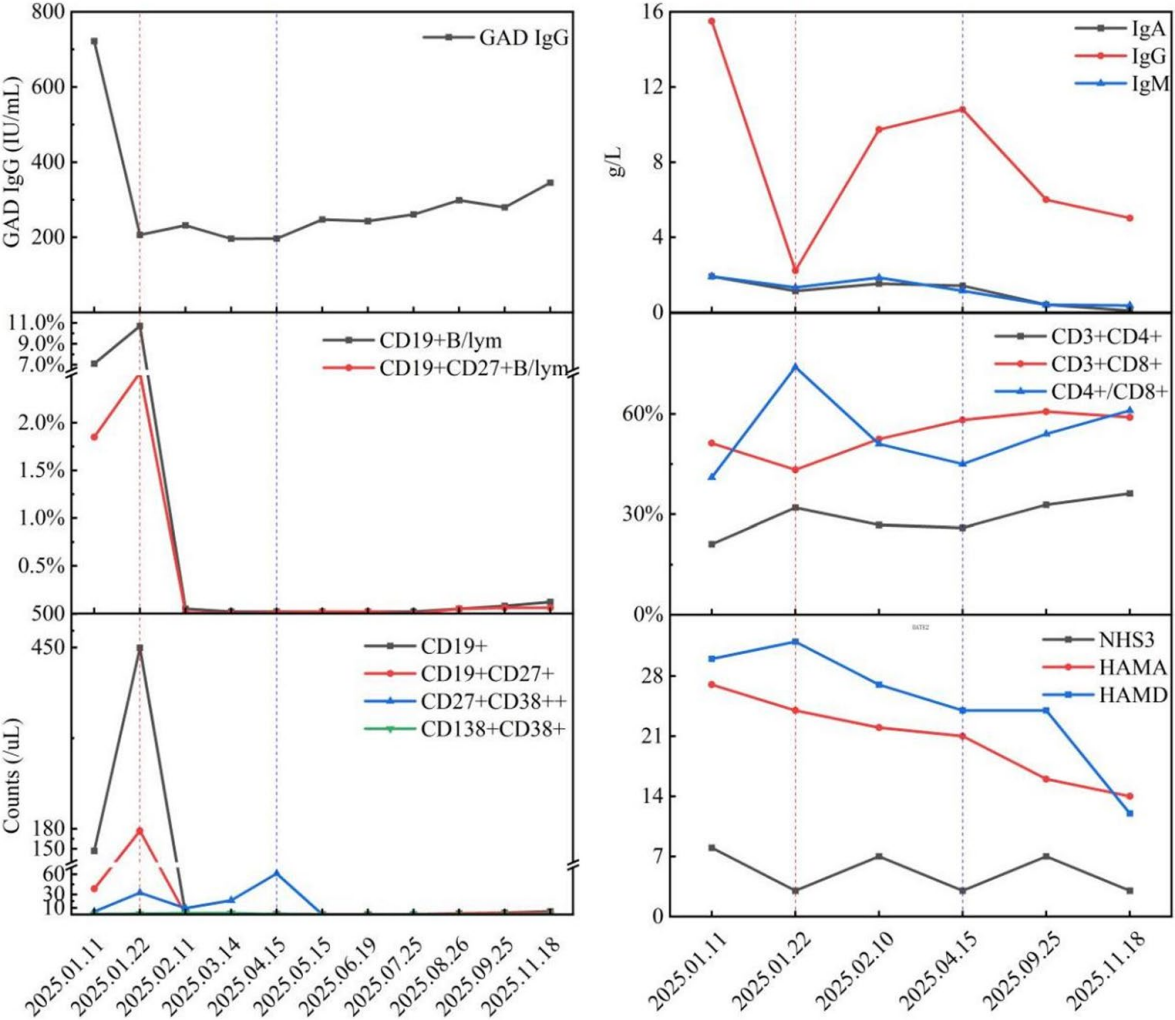
Longitudinal changes in serological and clinical parameters during sequential immunotherapy. The vertical red line marks the start of Rituximab therapy (following initial MP + IA treatment); the vertical blue line marks the start of Daratumumab therapy

During ongoing treatment with four antiepileptic agents and mycophenolate mofetil (0.25 g twice daily), intensified immunosuppression commenced on January, 2025. The regimen comprised intravenous methylprednisolone (80 mg daily) and protein A immunoadsorption (5 sessions on alternate days), resulting in a marked reduction in serum anti-GAD antibody levels to 206.3 IU/mL. Rituximab (600 mg) was subsequently administered to consolidate this response. Over the following 2 months, peripheral CD19^+^ B cell counts were depleted (fewer than 1 cell/µL). By April 2025, an increase in CD27^+^CD38^++^ plasmablast counts was observed, coinciding with a rise in seizure frequency to three episodes per month. As a result, Daratumumab, a CD38 targeted agent, was introduced with an induction dose of 8 mg/kg at weeks 0 and 2, followed by monthly maintenance at 4 mg/kg for a total of seven infusions. This regimen resulted in depletion of peripheral CD27^+^CD38^++^ plasmablasts and sustained CD19^+^B cell counts below 10 cells/µL (Fig. 4).

At the December 2025 follow-up, CSF anti-GAD65 antibody titers had decreased to 1:100 by CBA. Antinuclear antibodies were negative, anti-Ro-52 remained positive (++), and liver function tests had normalized. EEG continued to show moderate bilateral theta and delta waves. Brain MRI still demonstrated the same mild bilateral hippocampal atrophy. The patient currently experiences approximately two seizures per month, predominantly focal seizures with impaired awareness (NHS3 score: 3). Anxiety and depression have improved significantly (HAMA: 14; HAMD: 12). Although fully independent in daily activities, the patient has not yet returned to work.

## Discussion

This review provides an overview of reported cases of autoimmune encephalitis (AE) following transplantation, including bone marrow, hematopoietic stem cell, and solid organ transplants. Post-transplant AE predominantly affects females (11 of 14 cases) and typically manifests during the late phase, defined as more than 100 days after transplantation. The most common clinical manifestations are seizures, cognitive decline, psychiatric symptoms, ataxia, and movement disorders. Anti-NMDAR and anti-GAD65 antibodies are most frequently identified. First-line acute treatments generally include methylprednisolone (MP), IVIG, or plasma exchange, while some cases require additional immunosuppressive agents such as tacrolimus, cyclophosphamide, or RTX. Clinical outcomes are heterogeneous, ranging from complete remission to mortality (Table 1).

**Table 1:** Literature review of post-transplant autoimmune encephalitis

Time of report	Age	Sex	Initial disease	Transplantation history	Clinical characteristics	Autoimmune encephalitis antibodies	Immunology therapy	Outcome	Liturature
2008	31	F	IgG-kappa multiple myeloma (MM)	Auto-BMT; developed cGVHD	Stiff-person syndrom	Serum anti-GAD65 (104 nmol/L)	IVIG and MP	SPS symptoms persist; MM remains in remission.	[8]
2015	7	M	Idiopathic aplastic anemia	A matched-related donor BMT; developed cGVHD	Abnormal behavior, poor attention, cognitive deficits, abnormal movements	VGKC and LGI1	MP and IVIG	Significant improvement in behavior and neuropsychological testing and remained seizure-free	[9]
2018	54	F	Chronic pyelonephritis and end-stage renal disease	Renal transplantation; developed cGVHD	Episodic vertigo, diplopia, gait ataxia, personality change, fluctuating consciousness, orofacial dyskinesia, autonomic dysfunction	Serum and CSF anti-NMDAR	IV corticosteroids, IVIG and cyclophosphamide	Died from multi-organ failure.	[10]
2018	62	F	Acute myeloid leukemia	Allo-HCT from a matched sibling; developed cGVHD	Diplopia, gait disturbance, severe dysarthria, ataxia	Serum anti-GAD65 (8.7U/mL) and CSF anti-GAD65 (2.65 U/mL)	MP, IVIG and RTX	Died 4 months later.	[11]
2019	5	F	Pineoblastoma	HSCT; developed cGVHD	At 13 months post-HSCT: seizure clustering and altered mental status. At 27 months: status epilepticus and altered mental status	13 months post-HSCT: serum anti-GAD (65,100 U/mL). 27 months post-HSCT: serum anti-GAD (142,000 U/mL); CSF anti-GAD (238 U/mL).	IVIG, MP and plasma exchange	Mental status partially recovered; improved alertness, decreased seizures; no relapse for 5 years; unable to communicate or walk independently at age 15.	[12]
2019	69	F	Liver cirrhosis	Liver transplantation; developed cGVHD	Progressive mental impairment, aphasia, disorientation, generalized epileptic seizures, rapid progression to vegetative state	Serum anti-NMDAR (1:200) and CSF (1:100) (by CBA)	MP, IA, IVIG, RTX, and cyclophosphamide	Died 2 years after disease onset due to septicemia.	[13]
2019	17	M	Hodgkin lymphoma	Allo-BMT from brother after prior auto-BMT; developed aGVHD	Acutely altered mental status, insomnia, agitation, paranoia, suicidal/homicidal ideation	Negative.	MP, plasma exchange, RTX	Dramatic and sustained improvement in mental status; resolution of suicidal ideation; returned to neurologic baseline.	[14]
2020	58	M	Alcoholic cirrhosis	Liver transplantation; developed cGVHD	Confusion, lethargy and lightheadedness	Negative.	MP	Remission	[15]
2020	60	F	Acute lymphoblastic leukemia (ALL)	Allo-HSCT from a HLA-matched sibling donor; developed aGVHD	Short-term memory dysfunction, emotional lability, involuntary movements of left hand; loss of consciousness	CSF anti-GluR (anti-GluN2B & GluN1 peptide) positive.	MP and anti-thymocyte globulin	Died on day 76 post-transplantation.	[16]
2023	7	F	Shwachman-Diamond syndrome	Allo-HSCT from a 9/10 HLA-matched unrelated donor; developed cGVHD	Abnormal behavior, episodic oral twitching, and automatisms	Serum anti-mGluR5 ( 1:1000 by CBA)	IVIG and MP	Abnormal behaviors notably improved; EEG results improved.	[17]
2023	50	F	Primary hepatocellular carcinoma	Liver transplantation; developed cGVHD	Generalized convulsions and loss of consciousness	Serum anti-CASPR2 (titer 1:32 by CBA); CSF anti-CASPR2 (titer 1:3.2 by CBA)	IVIG	Improvement of clinical symptoms and EEG.	[18]
2024	57	F	Aplastic anemia	Allo-HSCT from her daughter; developed cGVHD	Cognitive dysfunction, memory loss, and involuntary movements	CSF anti-GFAP (titer 1:320 by CBA	MP and tacrolimus	Restoration of cognitive function	[19]
2024	66	F	Chronic glomerulonephritis, end-stage renal disease	Renal transplantation; developed cGVHD	Headaches, paraplegia, incontinence, abdominal pain, focal impaired awareness seizures	Serum anti-GlyR (titer 1:100 by CBA)	IVIG and MP	Symptoms eased noticeably, mental status restored. condition stable.	[20]
2025	32	F	Type 1 diabetes mellitus, end-stage renal disease	Simultaneous kidney-pancreas transplantation; developed aGVHD	Subacute altered mental status, persistent fevers, dysmetria, headache	Serum anti-GAD65 (95.4 nmol/L) and CSF (0.11 nmol/L)	MP, IVIG, ruxolitinib, tacrolimus	Died 6 months post-transplant	[21]

*aGVHD* Acute graft-versus-host disease, *EDSS* Expanded disability status scale, *ELISA* Enzyme-linked immunosorbent assay, *GABA* γ-aminobutyric acid, *GFAP* Glial fibrillary acidic protein, *GlyR* glycine receptor, *HLA* Human leukocyte antigen, *Allo-BMT* Allogeneic bone marrow transplantation

Unlike previously reported cases of cGVHD-mediated autoimmune encephalitis, this patient presented with insidiously chronic seizures beginning four years after allo-HSCT and persisting for seven years. Recent studies have emphasized the increasing recognition of immune-mediated epilepsy. Data show that approximately 5% of antibody-mediated focal epilepsy cases may remain undiagnosed [22]. In this case, initial antibody screening was not performed due to limited awareness of immune-mediated epilepsy. To clarify the etiology, autoantibody testing for autoimmune encephalitis was conducted, revealing positive anti-GAD65 antibody in both CSF (CBA, 1:320+) and serum (CBA, 1:3200+). Given the characteristic clinical features, he was diagnosed with definite anti-GAD65 antibody-associated epilepsy.

Notably, anti-GAD65 antibody-associated epilepsy may progress to autoimmune-associated epilepsy (AAE), which must be distinguished from acute symptomatic seizures secondary to autoimmune encephalitis (ASSAE). According to the 2020 International League Against Epilepsy (ILAE) conceptual framework, AAE is defined as persistent epilepsy following autoimmune encephalitis with limited responsiveness to immunotherapy [23]. In this case, the patient’s seizures persisted for seven years, required multiple antiseizure medications, and showed an incomplete response to immunotherapy, supporting classification as AAE rather than ASSAE.

Although anti-GAD65 antibody-associated epilepsy can occur independently, the presence of multiple clinical features aligns with the diagnostic criteria for presumptive CNS-GVHD established by the 2010 Consensus Conference on Clinical Practice in Chronic GVHD [4]: (1) prior history of cGVHD, (2) concurrent systemic autoimmunity (ANA, anti-Ro52 positivity, mild vitiligo and hepatic dysfunction), (3) absence of infectious, metabolic, or neoplastic causes after comprehensive evaluation, and (4) an immunotherapy-responsive course with a parallel reduction in antibody titers. However, definitive pathological confirmation of CNS-cGVHD was not available.

Considering the high risk associated with surgical intervention, an individualized immunotherapy regimen was recommended. The therapeutic approach followed a sequential strategy: initially, immune suppression was achieved using MP and rapid antibody clearance was facilitated through IA; Subsequently, RTX was administered to inhibit B cell maturation, differentiation into antibody-producing cells, antigen presentation, and cytokine secretion [24]; finally, anti-CD38 monoclonal antibody Daratumumab therapy was introduced in refractory cases to target plasmablasts and long-lived plasma cells that are resistant to previous interventions [25, 26]. The combination of RTX and Daratumumab led to the depletion of peripheral B cells and both short-lived and long-lived plasma cells, thereby potentially restricting antibody trafficking and limiting the migration of autoreactive B cells and plasma cells into the brain [26, 27].

Throughout the treatment course, the patient’s peripheral blood immune cell subsets, immunoglobulin levels, anti-GAD antibody titers, tumor markers, and STR chimerism were systematically monitored. Laboratory data demonstrated a reduction in peripheral IgA, IgG, and IgM levels following protein A IA and low-dose Daratumumab treatment. After IA, the serum anti-GAD antibody titer decreased to one-third of baseline, indicating effective autoantibody clearance. Analysis of peripheral T cell subsets revealed a higher proportion of CD8^+^ T cells than CD4^+^ T cells, resulting in a CD4^+^/CD8^+^ ratio less than 1. This ratio increased gradually during treatment with MP, IA, and Daratumumab, suggesting a favorable response to immunosuppressive therapy. Further observation through extended follow-up is necessary to determine whether the CD4^+^/CD8^+^ ratio correlates with long-term prognosis.

However, we acknowledge the limitations of this study. Due to the invasive nature of brain tissue biopsy, pathological verification of CNS-GVHD was not obtained. Antibody monitoring was primarily conducted using serum GAD antibody testing during monthly follow-ups, with CSF GAD65 antibody reassessed by CBA only at approximately one year after intensified immunotherapy—both of which showed marked declines. The patient continues on maintenance therapy with RTX and daratumumab, necessitating long-term follow-up to further evaluate the benefits and risks of treatment.

## Conclusion

In summary, we suggest active screening for autoimmune-associated epilepsy antibodies in patients in remission following allo-HSCT who develop insidious-onset seizures. A combined treatment regimen of MP, IA, RTX, and daratumumab resulted in a marked reduction in CSF anti-GAD65 antibody and serum GAD antibody titers, as well as effective control of the patient’s seizures and mood disorders. This case provides a valuable reference for the management of CNS-cGVHD.

## Data Availability

The original contributions presented in the study are included in the article. Further inquiries can be directed to the corresponding author.
